# Hybrid Routing, Modulation, Spectrum and Core Allocation Based on Mapping Scheme

**DOI:** 10.3390/s20216393

**Published:** 2020-11-09

**Authors:** Edson Rodrigues, Eduardo Cerqueira, Denis Rosário, Helder Oliveira

**Affiliations:** Federal University of Pará, Belém 66075-110, Brazil; cerqueira@ufpa.br (E.C.); denis@ufpa.br (D.R.); heldermay@ufpa.br (H.O.)

**Keywords:** Elastic Optical Network, space division multiplexing, routing

## Abstract

With the persistently growing popularity of internet traffic, telecom operators are forced to provide high-capacity, cost-efficient, and performance-adaptive connectivity solutions to fulfill the requirements and increase their returns. However, optical networks that make up the core of the Internet gradually reached physical transmission limits. In an attempt to provide new solutions emerged, the Space-Division Multiplexing Elastic Optical Network emerged as one of the best ways to deal with the network depletion. However, it is necessary to establish lightpaths using routing, modulation, spectrum, and core allocation (RMSCA) algorithms to establish connections in these networks. This article proposes a crosstalk-aware RMSCA algorithm that uses a multi-path and mapping scheme for improving resource allocation. The results show that the proposed algorithm decreases the blocking ratio by up to four orders of magnitude compared with other RMSCA algorithms in the literature.

## 1. Introduction

The constant growth in the number of Internet users and greater applications with high bandwidth demands have made the traffic passing through the backbone of the Internet increase exponentially in recent years [1]. Furthermore, it is expected that the traffic will continue to grow, pushing the current network to its physical limit [2]. Despite Wavelength-Division Multiplexing (WDM) technology meeting the demand for a period, it does not allow the spectrum of the network to be used in the best possible way due to its rigid coarse granularity, leading to a waste in spectrum caused by the size of requests that do not fit precisely to frequency slots [3]. To overcome the limitations of the transport data network that employs WDM, the Elastic Optical Network (EON) has been proposed, which has increased flexibility for the allocation of a bandwidth [4,5]. In EONs, the fixed spectrum is allocated in small units called slots, allowing flexible allocation to accommodate the connections’ bandwidth demands, grouping several of these units. In this way, the residual band is minimized, leading to more efficient use of the spectrum, and separating each lightpath established by a band called Filter Guard Band (FGB) [6].

To substantially expand the network’s physical capacity and increase the possibilities of data transport, the spacial strategy has been explored through the use of multi-core fibers, implementing Space-Division Multiplexing (SDM) technology to the EONs [7,8]. The increase in the flexibility and availability of resources, with the advent of SDM-EON technology, gives rise to new problems, which need to be solved for the network’s best use. One of these problems is related to the routing and allocation of resources. The addition of more cores on fiber increases the complexity in allocating resources as it adds a dimension to be considered when choosing paths for network requests. In SDM-EON, two constraints must be considered to establish a connection from a source to the destination: the continuity of frequency slots, which checks whether the requested slots are continuous in all links in the path, avoiding core switching, and contiguity, which ensures that all slots are allocated in contiguous bands of the spectrum same core [4].

When allocating resources in SDM-EON, in addition to the continuity and contiguity constraints, the transmission rate can also be considered, i.e., increasing the bit rate encoded by each optical symbol. The factor that limits the use of modulation formats is their sensitivity to losses, thus the greater the number of bits per symbol, the greater the probability of an error in its decoding [9]. This effect makes transmissions over a longer distance more complex due to losses caused by signal attenuation and interference. From this concept, the use of adaptive modulation in optical networks arose, able to regulate the number of bits per symbol to be used according to the distance to be traveled [9]. The greater the number of bits transmitted per symbol, the smaller the signal’s reach. The multi-core utilization and adaptive modulation bring a new problem to resource allocation for requests, the Routing, Modulation, Spectrum, and Core Allocation (RMSCA) problem [4].

Allocating requests that preserve continuity and contiguity conditions in a single path can be difficult when the traffic load is high. In allocating and releasing spaces, requests create small slices of bandwidth in the spectrum that are not enough for some requests, rejecting those that cannot be accommodated on the network. In an attempt to increase the number of connections established and improve the spectrum’s use, providing more than one path for a request presents good results compared to using only one path [10]. The use of multi-paths presents challenges known as the time of arrival of each path. To deal with this problem, the differential delay is added as one of the path selection conditions, where the difference in arrival time between the first and the last path must respect the acceptable limit [11].

This paper’s contribution is the introduction of an RMSCA algorithm called Hybrid Routing, Modulation, Spectrum and Core Allocation based on the Mapping Scheme in SDM-EONs (*PANORAMIC*). The proposed algorithm aims to reduce the number of blocked requests using hybrid routing and adaptive modulation. We call hybrid routing a policy, which first tries to allocate a single path for a request and then allocates multiple paths only if the allocation of a single path is impossible. The proposed algorithm uses the spectrum mapping scheme to find paths that can accommodate requests that use adaptive modulation. Finally, we simulated an online and dynamic scenario, which showed the benefits of the *PANORAMIC* in terms of blocking bandwidth and Energy Efficiency while keeping the crosstalk per slot and fragmentation at an acceptable level compared to state-of-the-art RMSCA algorithms. The results obtained here extend those found in preliminary investigation [12]. While previous work [12] proposes an RMSCA algorithm using single routing, we propose an RMSCA algorithm using hybrid routing in this paper. The results present that the proposed hybrid lightpath provisioning mechanism outperforms the traditional single-path routing by reducing the blocking ratio while reducing traffic that may be affected by single link failures.

This paper is organized as follows. In Section 2, we give an overview of the related work. Section 3 presents the networks used and its physical features and describes how the algorithm works. Section 4 is the performance evaluation for the algorithm. Section 5 presents the conclusions and future works.

## 2. Related Work

The space-division multiplexing elastic optical networks have driven several studies to increase the allocation of resources. However, to the best of our knowledge, this is the first work that considered multi-path routing and delay differentials to solve the routing, modulation, spectrum, and core allocation problem in SDM-EON.

Zhu et al. [13] propose a dynamic Routing, Modulation Level, and Spectrum Assignment (RMLSA) based on both online and offline path computation with several path selection policies. The provisioning resources use a hybrid single-/multi-path routing scheme. However, they do not consider delay differential, which may lead to an unreal case where the network can support any multi-path. Moreover, the article is not concerned with the increasing current traffic and consequently does not consider fibers with multiple cores.

Monghaddam et al. [14] present a Mixed Integer Linear Programming (MILP) and a heuristic algorithm for dealing with the crosstalk-aware RMCSA and scheduling problem. In their scenario, they consider static traffic, and paths are calculated in advance using the K-shortest path algorithm. The MILP makes sure that only one single path is assigned for each call, then adaptive modulation is applied, and the strict crosstalk for each slot in the path is calculated. Then, the maximum modulation is selected if the crosstalk threshold allows the connection without any signal degradation. The objective is to reduce the resources used by reducing the number of slots allocated. The heuristic equilibrates the load on links as the use of frequency slices index. Despite using the spatial dimension, they do not consider the multi-path as a strategy to increase the number of acceptances and reduce the waste in the spectrum and use a spectrum scan that does not favor the best use of the spectrum.

Yin et al. [15] introduce a survivable crosstalk-aware multi-path strategy in SDM-EONs considering multi-path protection. They used the super-channels approach, utilizing cost-saving by reducing the number of required slots lasers when the transponders architecture of spatial super-channel. They used Bhandari’s disjoint path algorithm to calculate multiple routes in advance. Then, they verified that a connection can be established, in case the crosstalk for the path is acceptable. However, the authors disregard differential delay and multiple modulation formats to transmission, causing a spectrum wastage.

Oliveira and da Fonseca [16] introduce an algorithm that provides paths exclusively when a protection path can also be found. For such a case, they adopt the single -ath strategy at first, then, in the case where a single path cannot be allocated, the adoption of multi-paths happens. In both cases, Shared Backup Path Protection (SBPP) is utilized to guarantee the network’s survivability. They use Dijkstra’s algorithm to find the shortest path for both single- and multi-path cases, but they do not consider adaptive modulation, utilizing only one modulation format, which leads to spectrum underutilization, contributing to increasing the blocking probability.

Yousefi et al. [17] introduce two state-of-the-art novel metrics: holding time and coefficient of the variant metric. Based on these metrics, they propose three algorithms to solve the fragmentation problem and improve blocking probability in the Routing, Spectrum, and Core Allocation (RSCA) problem. The proposed algorithms utilize the spectrum waste base algorithm to find a suitable rectangle for each connection to fit the K-Shortest Path, considering the fragmentation in each algorithm in order to reduce the fragmentation ratio. Adaptive modulation and multi-path are not used in algorithms proposed by the authors.

Yousefi et al. [18] introduce six algorithms aiming to solve the fragmentation problem and improve blocking probability, dividing the connection into multiple paths. The algorithms consider External Fragmentation Metric and splitting of lightpaths to reduce blocking probability. When fragmentation is not an obstacle, a connection may be established if the K-shortest path algorithm finds an available path. They use the FindFirstFreeGap algorithm to allocate frequency slots to a request. Their algorithms work for Routing and Spectrum Assignment (RSA) for dynamic scenarios, but the RMSCA problem cannot be supported because they do not use any core or adaptive modulation.

Yousefi and Rahbar [19] introduce three novel algorithms to solve fragmentation problems and improve the blocking probability for SDM-EON. The algorithms can reduce blocking probability by controlling fragmentation in cores, since they try different ways of assigning a spectrum for a connection. However, they only consider the RSCA problem without considering different modulations, and only one algorithm utilizes a multi-path mechanism, but differential delay constraint is neglected.

Yousefi and Rahbar [20] develop three algorithms—MINCROSS, MINFRAG, and MODFRAGCROSS—to solve crosstalk and maintain the physical layer security level and reduce blocking probability. The authors consider the impairment and physical layer security leak attack caused by inter-core crosstalk. They try to control blocking probability by preventing fragmentation and crosstalk and used an algorithm called ExactFit to find a free gap that can allocate the request utilizing the exact number of frequency slots. In MINCROSS, they prioritize the crosstalk as the main condition when selecting the path, while in MINFRAG they consider fragmentation as first conditional. In MODFRAGCROSS, they keep both crosstalk and fragmentation at acceptable levels. Although the work considers crosstalk per-slot and fragmentation metrics, they do not apply modulation and multi-path, so the spectrum is not used in the best way.

Zhu et al. [21] propose a multi-path, fragmentation-aware routing modulation and spectrum assignment for advance and immediate reservations. The authors proposed splitting advance and immediate reservation requests into smaller parts, transferring each of these parts along with single- or multi-paths, taking into account the delay differential addressing with additional electronic buffering in the higher layer of the destination node. They propose a time and spectrum fragmentation occurrence measurement. They applied adaptive modulation but did not use a multi-core fiber.

Moghaddam et al. [22] formulate a mixed-integer, linear programming MILP in networks with dedicated and shared path protection schemes. A heuristic algorithm to solve a large scale problem is also proposed. The proposed algorithms support the network with both dedicated and shared path protection and consider the crosstalk strictly. The K-shortest path calculates the path between two points with adaptive modulation, but the multi-path mechanism is not utilized in this approach.

Moura and Da Fonseca [23] propose four RMSCA algorithms based on image processing algorithms. They utilize two image processing algorithms—Connected Component Labeling (CCL) and Inscribed Rectangles Algorithm (IRA)—-to reduce computational complexity. For ImageRCMLSA, CCL was used combined with the best fit policy for reducing spectrum waste. Modulations are calculated by the second of the transmission distance and the estimated crosstalk, and then a fitting policy is applied. However, requests that cannot find a single lightpath are not allowed to split into paths to find available paths.

Dharmaweera et al. [24] present an analytical optimization formulation for small networks and a heuristic for large networks to increase spectral efficiency. Both use the multi-path technique to allocate requests that could not find a single path from point to another, and also apply traffic grooming to avoid the waste caused by the number of guard bands utilized in optical connections. The authors use the Yen Shortest Path algorithm to find the path to calls. However, they did not consider adaptive modulations during the calculation between source to destination.

Malekzadeh and Shahkooh [25] introduce an ILP and a heuristic to routing, modulation, and spectrum allocation in EONs. In ILP, they aim to minimize the total spectrum utilized in the network, utilizing the K-shortest path algorithm to find the shortest path. The proposed heuristic uses the single- and multi-path routing. A threshold to calls with multi-path is set and, if this limit is passed, then the request is blocked. However, they did not consider the space dimension in their work.

Table 1 compares the algorithm proposed in this paper to various others described in the literature concerning the use of space-division multiplexing, the use of adaptive modulation, the employment of the multi-path, and consideration of the delay differential. Based on our analysis of the state-of-the-art, we conclude that few studies consider the study of delay differential when multi-path is used. To the best of our knowledge, this is the first article to solve the routing, modulation, spectrum, and core allocation problem through multi-path considering the differential delay.

## 3. Algorithm

This section introduces Hybrid Routing, Modulation, Spectrum, and Core Allocation based on Mapping Scheme (*PANORAMIC*). The algorithm aims to deal with the RMLSCA problem, utilizing a mapping scheme that verifies the topology with all frequency slots in each core, trying to find a free gap capable of fitting a request. When the requirements are met, the connection is established. In the routing process, modulation formats are chosen according to the transmission distance, allowing signal decoding.

### 3.1. Network and System Model

We adopted an optical network operating with a spatially flexible reconfigurable optical add/drop multiplexers capable of a wavelength-selective switch, and space-wavelength granularity, with Multiple-Input Multiple-Output (MIMO) transceivers. Furthermore, we consider Multi-Flow Transponders (MFTs) to split into multiple sub transponders to facilitate allocation.

The network topology is composed of bidirectional fibers with seven cores arranged in a hexagonal array. Each of these cores has 320 slices of frequency slots with a transmission capacity of 12.5 Gbps. We represent the network model considering actual distances from one point to another. We kept the continuity and contiguity restrictions, which means that the equipment does not allow core- and slots-switching to occur during the transmission. The calculation of the number of slots required for transmission is done based on the number of bits per symbol that the modulation applied to the transmission can transmit. Thus, the greater the modulation, the greater the amount of data transmitted in a slot, reducing the total number of slots used for transmission. In addition, a slot is always allocated for the FGB, which separates the transmissions.

Decoding the signal is essential for the transmission to be carried out successfully. Thus, several modulations were used in order to improve the use of the network spectrum. However, the modulation choice must be made, so that the maximum distance that the signal can arrive with quality that can be read. The most efficient modulation level for the transmission must be selected according to its characteristics [26]. Table 2 shows the modulation characteristics considered in our network scenario.

### 3.2. Algorithm  *PANORAMIC* 

*PANORAMIC* is an RMLSCA algorithm that performs in SDM-EONs, with different loads, scenarios, and topologies. The algorithm’s objective is to increase the number of connections established in the network and improve the use of spectrum, avoiding the waste of frequency slots that can be idle after several connections and disconnections. The algorithm does not switch cores during the transmission, to prevent signal conversion from optical to electrical, keeping the continuity and contiguity restrictions. The Mapping Scheme used in our proposal offers a map of the available resources in the network, leading to an increase in the number of paths. The algorithm receives the network’s traffic as the input and, after running the routing, returns the possible lightpath with sufficient resources to accommodate requests. Table 3 shows the notation that will be used to describe the algorithm.

Algorithm 1 receives a request and, with information such as source node, destination node, and bandwidth, then starts the routing process. Although it is a hybrid routing and multiple paths can be used, the PANORAMIC first looks for a single path in the network that can support the transmission. In Line 1, the algorithm is tested for each modulation level, then the number of sufficient frequency slots is calculated based on the number of bits that the modulation can transmit (Line 2). Then, in Line 3, the mapping algorithm is called for, and the number of frequency slots required is used as a parameter for the mapping, which return a set of graphs generated, which are essential to the process of finding the shortest available path for transmission. If the path is found, the connection is accepted and, soon after, established. Otherwise, a similar process is taken to find multi-paths for the connection. For the multi-path, modulations are tested in the previous process for a single path, from the highest to the lowest. The data are then divided into two, and the mapping takes place for each of the blocks to be transmitted. The mapping algorithm checks the spectrum of the network and returns the available spaces capable of supporting the transmission. Then, the difference in time of arrival of the paths is calculated and, according to what is established in [27], the difference between the paths cannot be greater than 15 ms, or 3000 Km, thus, if the difference between the paths is greater than the upper-bound, the connection is not accepted.

**Algorithm 1:** PANORAMIC

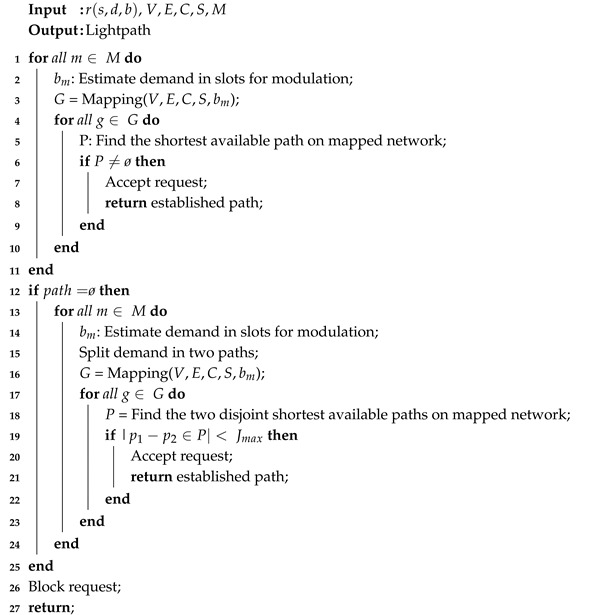



**Figure 1:**
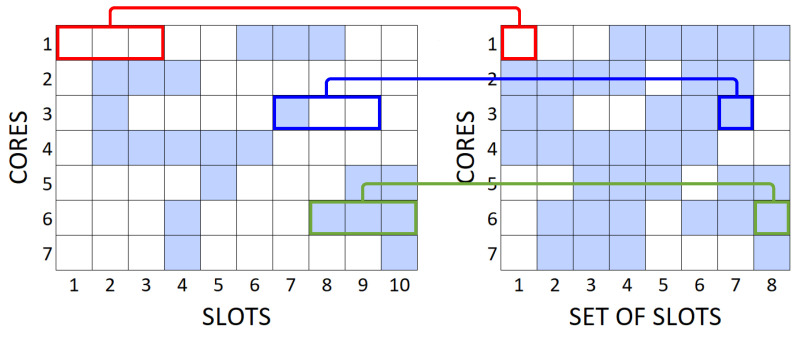
Spectrum Matrix and Mapped Matrix.

In the Mapping Algorithm (Algorithm 2), the network spectrum is mapped in a binary matrix format, where contiguous slots are scanned and, if they can accommodate the request, the initial index is mapped as available. Figure 1 represents three states of the mapping for a specific link (Line 1), with core-to-core sequence mapping (Line 2). First, represented in red, the block of slots required for the requisition is checked from left to right (Line 3) and, as all contiguous slots are available, that is, these slots can accommodate the requisition, it is represented in the mapping as available in Line 6. In the second case, represented in blue, only two of the three required slots are available in the main matrix, so this set is represented as unavailable (Line 9) in the mapped matrix, signaling that, although some slots are still available, they do not fully support the request. The third case, represented in green, shows where all the slots are occupied, so this set is mapped in the matrix as unavailable.
**Algorithm 2:** MAPPING
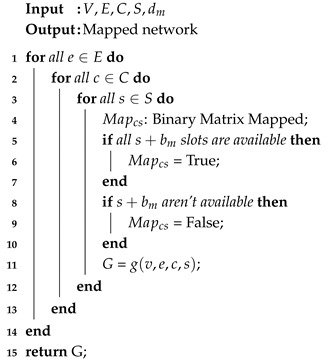


**Figure 2:**
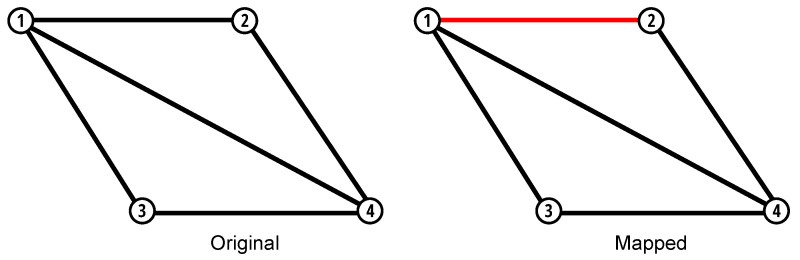
Mapped Graph.

Then in Line 11, for each of the mapped slots, a different graph is created to indicate the links where the core and slots continuity can be obeyed, this way, when a mapped matrix slot is unavailable, the generated graph does not recognize that mapped link as part of the topology, represented in Figure 2 by the red link. The set of graphs generated return to the main algorithm in Line 15, where the choice of the shortest path for transmission will be made, causing only the available paths to be found by the algorithm.

The complexity of the *PANORAMIC* algorithm is analyzed as follows: The complexity of mapping the network is O(E+V). For a single path, in the worst case, the chosen modulation will be the one with the lowest spectral efficiency, with it being necessary to transform the multigraph into graphs *M* times. In the worst case, to find the path, the Dijkstra algorithm runs *M* times in $C\times (N-b_{m})$ graphs, where Dijkstra’s amortized complexity is O(E+VlogV), obtaining a complexity of O(E+VlogV). The algorithm will not find a single path in the worst case, making it necessary to find a multipath. For this, the Suurballe algorithm is run *M* times in $C\times (N-b_{m})$ graphs, where Suurballe complexity is O(E+VlogV). As *M*, *N*, *C* and $b_{m}$ can be expressed as constants where the complexity of the *PANORAMIC* algorithm is O(E+VlogV).

## 4. Evaluation Performance

In this section, we present the evaluation for the proposed algorithm *PANORAMIC*, compared to other works in the literature with simulation parameters, with metrics involved at the evaluation for different scenarios.

### 4.1. Simulation Description and Metrics

**Figure 3:**
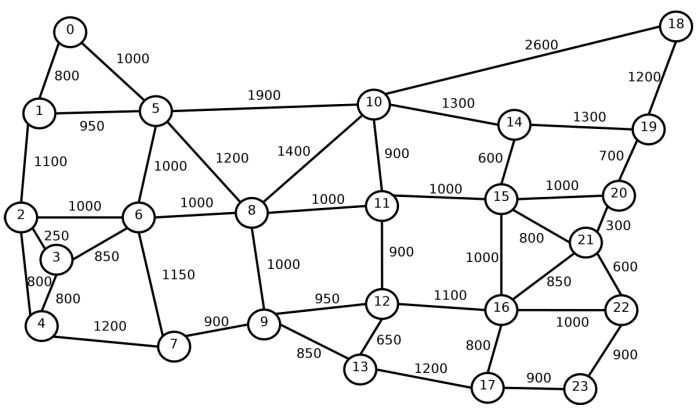
USA Topology.

**Figure 4:**
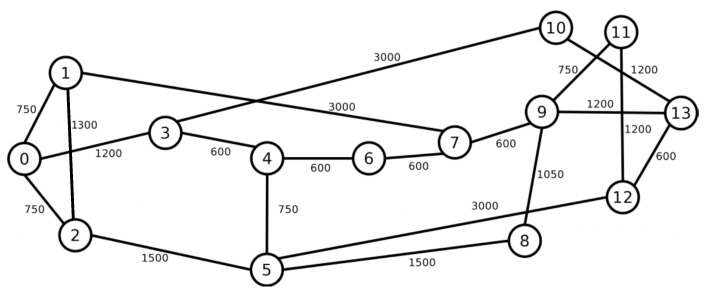
NSF Topology.

To assess the performance of the RMSCA algorithms, simulation experiments were conducted employing the FlexGridSim [28]. Requests followed a Poisson process and were uniformly distributed between all pairs of nodes. At least ten replications were generated for each scenario. We varied the network load from 50 to 1000 erlangs, and we used 100,000 connection requests in each simulation. Confidence intervals were derived using the independent replication method, and a 95% confidence level was adopted. Simulations for the different algorithms used the same set of seeds. Seven types of requests were employed by 25/50/125/200/500/750/1000 Gbps. The used fiber is composed of seven cores, and each fiber core was divided into 320 slots. The topologies used in the simulations were the USA (Figure 3), and NSF (Figure 4). The USA topology has 24 nodes, and 43 links and the NSF topology has 14 nodes and 19 links. The numbers on the links represent the length of the link in kilometers.

The metrics considered for evaluation of the algorithms are the Bandwidth Blocking Ratio (BBR), the Crosstalk per Slot (CpS), Energy Efficiency (EE), Fragmentation Ratio, and Modulation Format Percentage (MFP). The BBR value is calculated by the sum of all blocked bandwidth (α) during the simulation period, divided by the sum of all requested bandwidth (β) over the same period. The *BBR* is denoted by Equation (1).

(1) $$BBR=\frac{\sum \alpha }{\sum \beta }$$

The *CpS* is the result of the division between the number of allocated slots that have the same frequency (γ), both in the core being considered and in the adjacent cores of a link, and the total number of slots used in the same link (φ), calculated by periodic divisions (*T*) over periods of the same interval. *CpS* is denoted by Equation (2).

(2) $$CpS=\frac{\sum \frac{\sum \gamma }{\sum \varphi }}{T}$$

The *EE* calculation is made by dividing the sum of all bandwidth values (in Mbps) of the requests accepted in the network (γ), by the sum of the energy consumption values (in Joules) of the transponders, switches and optical amplifiers present in the routes of each accepted request (ϵ). The *EE* is denoted by Equation (3).

(3) $$EE=\frac{\sum \gamma }{\sum \epsilon }$$

The *FR* calculation is made by the maximum number of contiguous slots available (μ) to the number of available slots in the link (δ). The *FR* is denoted by Equation

(4) $$FR=\frac{Max(\sum \mu )}{\sum \delta }$$

The *MFP* is calculated by incrementing a counter for each time the modulation was used in a transmission ($T_{m}$); in the case of using multi-path, it was added for each of the disjoint paths. In the end, the number of times each modulation was used was divided by the total number of transmissions (Total) in single or multi-path. The *MFP* is denoted by Equation

(5) $$MFP=\frac{T_{m}}{Total}$$

### 4.2. Simulation Results

In this subsection’s figures, the curves labeled *PANORAMIC* show the results generated by the *PANORAMIC* algorithm proposed in this article. The curves with the label MINFRAG present the results obtained using the algorithm proposed in [20]. The curves labeled MINCROSS illustrate the results regarding the use of the algorithm proposed in [20]. The curves labeled ImageRCMLSA present the results regarding the use of the algorithm proposed in [23].

**Figure 5:**
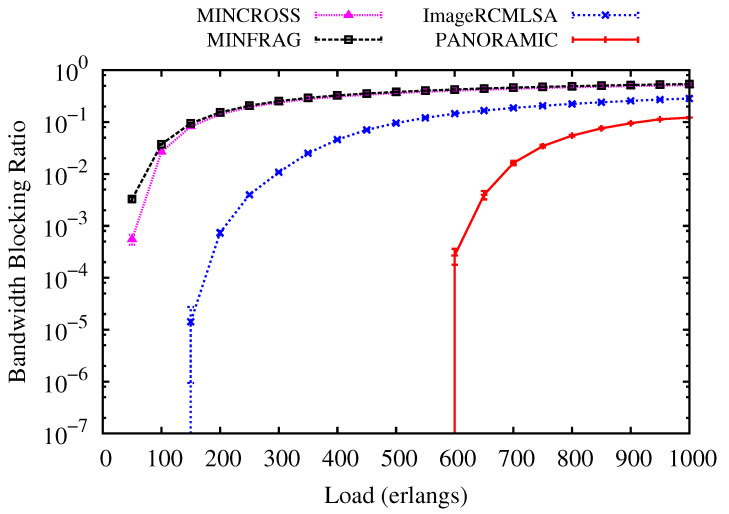
Bandwidth Blocking for USA topology.

**Figure 6:**
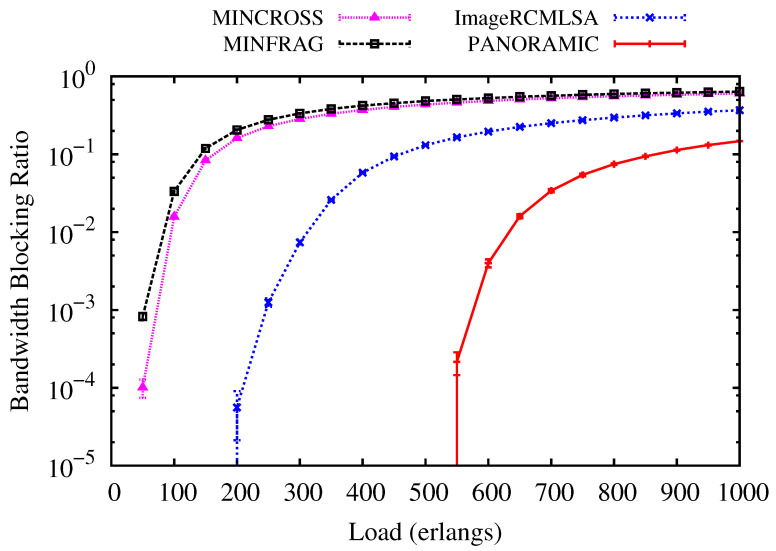
Bandwidth Blocking for NSF topology.

Figure 5 and Figure 6 present the BBR for USA and NSF topologies. For USA topology (Figure 3), while the *PANORAMIC* algorithm starts blocking requests only under loads of 600 erlangs, the ImageRCMLSA, MINCROSS and MINFRAG algorithms start blocking requests of about 150, 50 and 50 erlangs, respectively. For NSF topology (Figure 4), *PANORAMIC* algorithm starts blocking requests under loads of 525 erlangs, performing better than ImageRCMLSA, MINFRAG, and MINCROSS algorithms. While ImageRCMLSA starts blocking requests under loads of 200 erlangs, MINFRAG and MINCROSS algorithms start blocking requests under 50 erlangs. The results shown emphasize the efficiency of the mapping scheme used in the *PANORAMIC* algorithm. The mapping increases the number of free paths found in the network compared to the method used in ImageRCMLSA, MINCROSS, and MINFRAG, which offers a simpler finding path model. The K-Shortest Path (KSP) algorithm used in these algorithms considers the whole network, not only the free paths, taking the situation where the path found is already occupied. This means that, despite the network congestion, our algorithm still accommodates requests by allowing the demand to be divided into multiple-paths, where before it would not be possible to transmit using a single path, increasing the number of accepted requests as well as the quantity transmitted data, in addition to enhancing the efficiency of spectral use. However, due to the high number of connections established on the network, the interference caused by crosstalk tends to increase, but proposed algorithm has satisfactory results, as can be seen later. Topology USA has high connectivity between nodes, which favors the use of the disjoint paths strategy, while NSF Topology does not have this characteristic. However, the results showed similar curves for both topologies.

**Figure 7:**
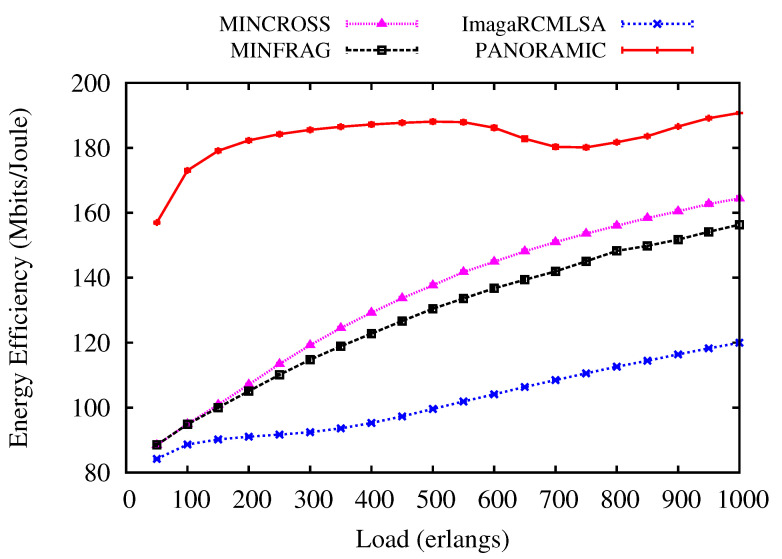
Energy Efficiency for USA topology.

**Figure 8:**
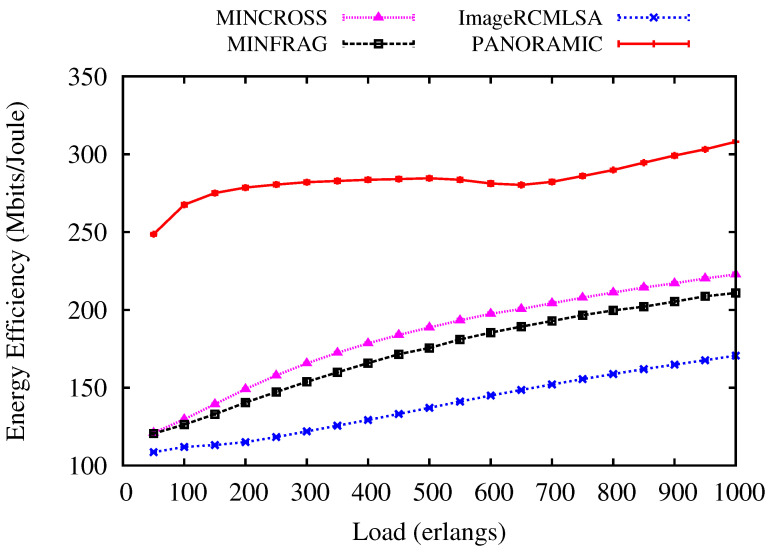
Energy Efficiency for NSF topology.

Figure 7 and Figure 8 show an evaluation of the Energy Efficiency performed by *PANORAMIC*, ImageRCMLSA, MINCROSS, and MINFRAG for NSF and USA topologies. Comparatively, the USA topology has a greater number of nodes than the NSF topology, thus the energy consumption is different, interfering in the energy efficiency for transmissions. For NSF topology (Figure 8), the *PANORAMIC* algorithm produces more energy efficiency than the compared algorithm under all loads simulated, demonstrating that the effort to make the connection makes the total data transmitted worthwhile. For USA topology (Figure 7), the *PANORAMIC* algorithm also has a better performance than ImageRCMLSA, MINCROSS, and MINFRAG. The energy efficiency is higher under all loads where algorithms were performed. However, when it comes to higher loads, the energy efficiency of all algorithms shows closer results.

**Figure 9:**
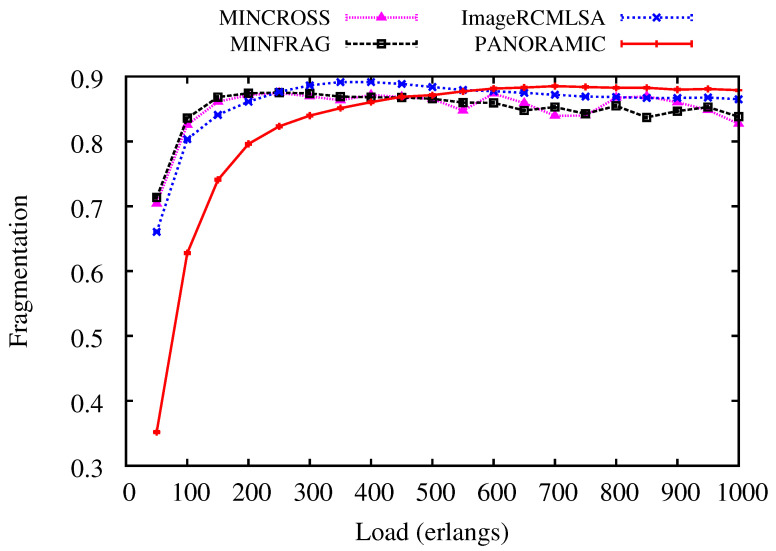
Fragmentation Ratio for NSF topology.

**Figure 10:**
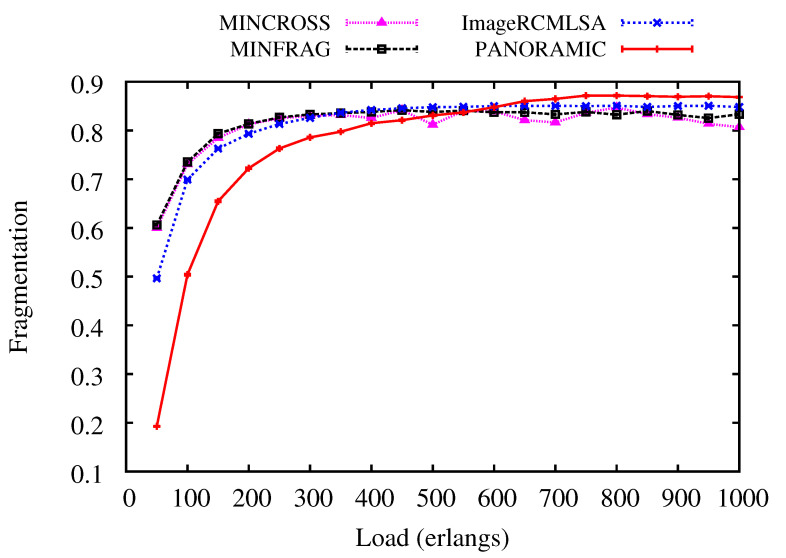
Fragmentation Ratio for USA topology.

Figure 9 and Figure 10 show results for Fragmentation to all simulated algorithms for NSF and USA topologies. For NSF topology, the *PANORAMIC* algorithm presents lower fragmentation than the compared algorithm up to 400 erlangs. Then, the results are very similar for all algorithms. However, ImageRCMLSA keeps fragmentation, with intermediate values between the algorithms used, while MINCROSS and MINFRAG present the lowest fragmentation, respectively. For USA topology, fragmentation for *PANORAMIC* has better results up to 450 erlangs, then values keep close, and under higher loads, the *PANORAMIC* algorithm presents the highest fragmentation among all algorithms. ImageRCMLSA presents acceptable results for fragmentation since it is not the main objective of this algorithm. The MINCROSS and MINFRAG algorithms present the best results for fragmentation, reaching both algorithms’ proposal, which deals with fragmentation control at some level of routing. When comparing the different topologies, the curve is very similar, concluding that there was no such big difference due to the physical characteristics of the topologies.

**Figure 11:**
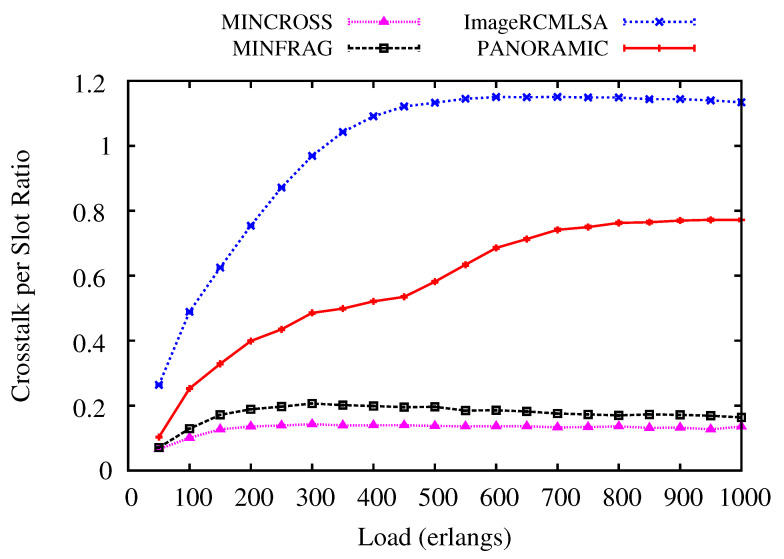
Crosstalk per Slot for NSF topology.

**Figure 12:**
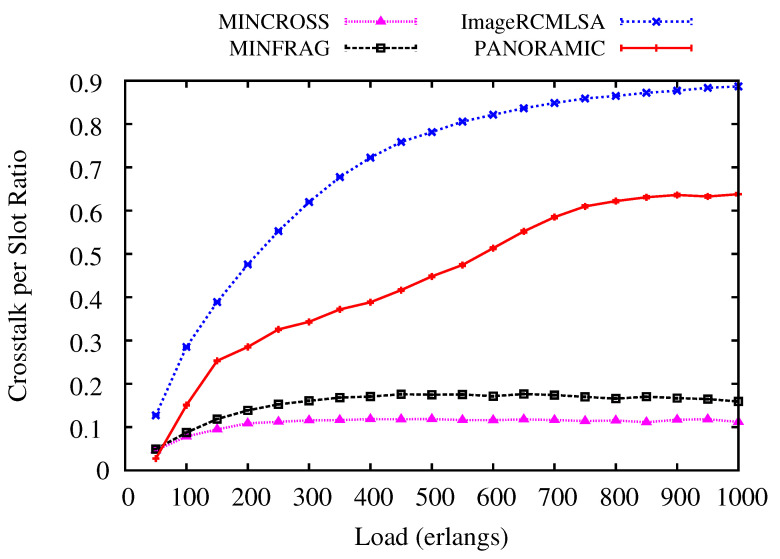
Crosstalk per Slot for USA topology.

Figure 11 and Figure 12 illustrate the Crosstalk per Slot (CpS) values analyzed for NSF and USA topologies. For NSF topology (Figure 11), the *PANORAMIC* algorithm has constant values for all loads simulated, keeping results between the values for the ImageRCMLSA, which is the algorithm that has higher values for CpS, and two of the algorithms compared MINCROSS and MINFRAG. As proposed, the MINCROSS algorithm archives the lowest CpS between all algorithms, and the MINFRAG presents higher CpS than MINCROSS and lower than *PANORAMIC*.

For USA topology (Figure 12), the results are very similar to obtained for NSF topology. ImageRCMLSA presents the highest CpS during all simulated loads, followed by the *PANORAMIC* algorithm, with medium values between the compared algorithms, and the MINFRAG and MINCROSS algorithm, respectively. These results show that MINCROSS is the most effective to reduce CpS between all algorithms. However, two factors may be caused by this: the goal of the algorithm is to reduce CpS and this algorithm blocks many requests, which implies that there are not as many established connections in the network as in the other algorithms, which means that there is little interference between cores. The ImageRCMLSA algorithm presents the highest CpS because of the way the gap of frequency slots for each request is selected, allowing the connection to allocate spaces in adjacent cores, which leads to more interference. Our algorithm’s goal is to accept a satisfactory number of requests and still keep an acceptable value of CpS.

**Figure 13:**
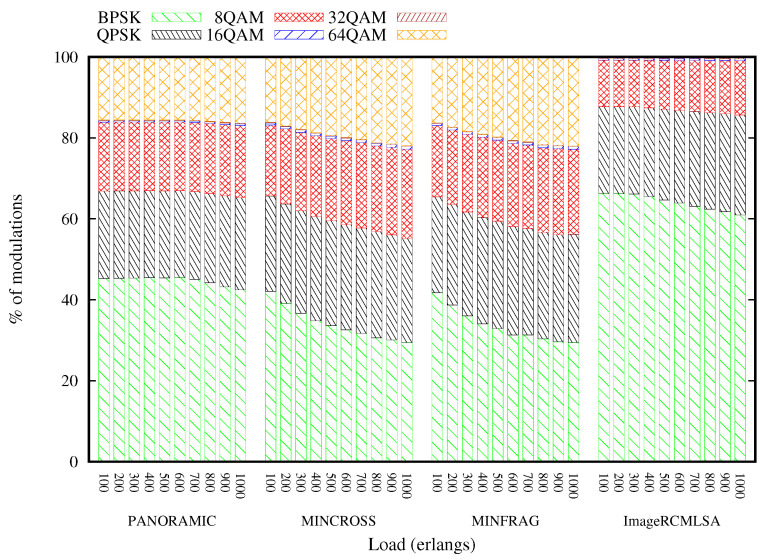
Modulation Format Percentage for USA topology.

**Figure 14:**
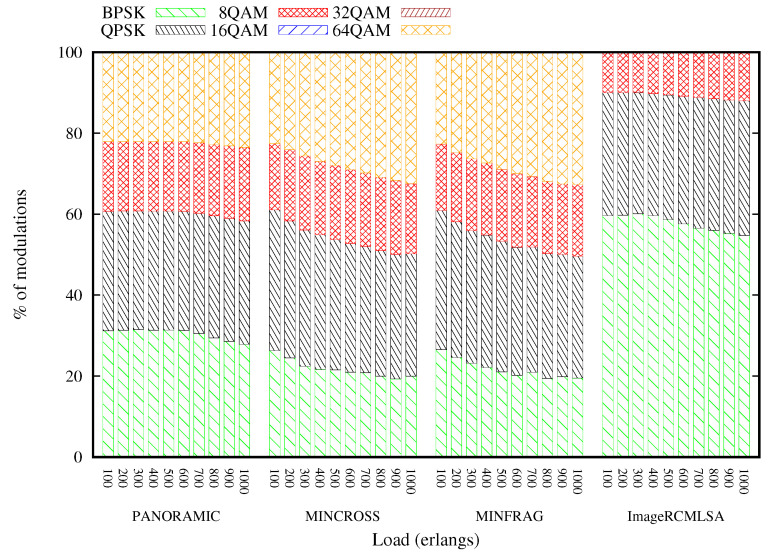
Modulation Format Percentage for NSF topology.

Figure 13 and Figure 14 present the arrangement of modulations used for each of the executed algorithms. For the Topology USA, the BPSK modulation is more used than the other modulations, representing more than 30% of the requests accepted in all loads of all algorithms. The USA topology has several connections, which implies a greater number of more distant transmissions. As the applied modulation is chosen, mainly based on the transmission distance, the BPSK modulation is predominant. In contrast, the 16 and 32QAM modulations reach intermediate distances, and therefore contemplate a small portion of the total transmissions with less than 2% in all USA topology situations. The modulations are slightly more distributed for the NSF topology, with QPSK and 64QAM modulations representing a large portion of the modulations. The NSF topology does not have as many links as the USA topology, and the distance between the nodes is average. Due to these network characteristics, modulations 16QAM and 32QAM were little used, representing less than 1% of accepted requests.

## 5. Conclusions and Future Work

This article presented *PANORAMIC*, an RMSCA algorithm that uses adaptive modulation with a spectrum mapping scheme capable of recognizing gaps of free frequency slots and using the hybrid single/multi-path technique for greater resource allocation. The *PANORAMIC* algorithm aims to reduce the bandwidth blocking rate by increasing the number of requests accepted by the network, in addition to improving the use of the network spectrum by reducing the number of idle frequency slots. For this, the *PANORAMIC* algorithm implements the multi-path technique for rejected requests at the first moment, therefore, for those requests that could not be allocated in a single path, the multi-path is sought in order to provide resources for the call, always considering the limitations generated by the differential delay caused by the possible difference in distance between the allocated paths. The *PANORAMIC* algorithm proved to be efficient in reducing the bandwidth blocking rate by accepting more requests, as well as promoting an improvement in energy efficiency for both network topologies used through the use of adaptive modulation, which increases the amount of data transmitted by used slots. In addition, the proposed algorithm also shows good results for the crosstalk rate per slot when comparing the number of connections established in the network with the other algorithms. As a future work, we aim to develop a machine learning model that considers the crosstalk and the spectrum fragmentation to improve resource allocation.

## Figures and Tables

**Table 1:** Related works comparison with the *PANORAMIC* algorithm.

Approach	SDM	Modulation	Multi-path	Delay Differential
Zhu et al. [13]		✓	✓	
Moghaddam et al. [14]	✓	✓		
Yin et al. [15]	✓		✓	
Oliveira and da Fonseca [16]	✓		✓	
Yousefi et al. [17]	✓			
Yousefi et al. [18]			✓	
Yousefi and Rahbar [19]	✓		✓	
Yousefi and Rahbar [20]	✓			
Zhu et al. [21]		✓	✓	✓
Moghaddam et al. [22]	✓	✓		
Moura and Da Fonseca [23]	✓	✓
Dharmaweera et al. [24]	✓		✓	✓
Malekzadeh and Shahkooh [25]		✓	✓	
*PANORAMIC*	✓	✓	✓	✓

**Table 2:** Modulation Characteristics.

Modulation Level	# Bits Per Symbol	Slot Capacity (Gb/s)	Maximum Distance (km)
64QAM	6	75	125
32QAM	5	62.5	250
16QAM	4	50	500
8QAM	3	37.5	1000
QPSK	2	25	2000
BPSK	1	12.5	4000

**Table 3:** Notation.

Notation	Definition
*s*	Source node
*d*	Destination node
*b*	Demand in slots
e∈E	Link from the network
v∈V	Node from the network
c∈C	Core from the network
s∈S	Slot from the network
r(s,d,b)	Request from *s* to *d* with bandwidth demand of *b*
m∈M	The set of modulations M={1,2,3,4,5,6} according to the Table 2
g(v,e,c,s)	A virtual graph that maps the slot *s* across the network
G={g(v,e,c,s)}	The set of virtual graphs
p∈P	Path for each request, whether single or multi-path
$J_{max}$	Delay Differential Threshold
$Map_{cs}$	Map Matrix for each fiber link
$b_{m}$	Number of slots required for transmission according to the modulation applied.

## Abbreviations

The following abbreviations are used in this manuscript:

BBR	Bandwidth Blocking Ratio
CCL	Connected Component Labeling
CpS	Crosstalk per Slot
EE	Energy Efficiency
EON	Elastic Optical Network
FGB	FilterGuard Band
FR	Fragmentation Ratio
IRA	Inscribed Rectangles Algorithm
MCF	Multi-Core Fiber
MFP	Modulation Format Percentage
MILP	Mixed Integer Linear Programming
MIMO	Multiple-Input Multiple-Output
PANORAMIC	Hybrid Routing, Modulation, Spectrum ANd CORe Allocation BAsed on Mapping SCheme
RSA	Routing and Spectrum Assignment
RMLSA	Routing, Modulation Level and Spectrum Assigment
RMSCA	Routing, Modulation, Spectrum and Core Allocation
RSCA	Routing, Spectrum and Core Allocation
RWA	Routing and Wavelength Allocation
SBPP	Shared Backup Path Protection
SDM	Space-Division Multiplexing
SDM-EON	Space-Division Multiplexing Elastic Optical Network
WDM	Wavelength Division Multiplexing
